# Simvastatin inhibits ischemia/reperfusion injury-induced apoptosis of retinal cells via downregulation of the tumor necrosis factor-α/nuclear factor-κB pathway

**DOI:** 10.3892/ijmm.2015.2244

**Published:** 2015-06-11

**Authors:** YU ZHANG, ZHUHONG ZHANG, HUA YAN

**Affiliations:** Department of Ophthalmology, Tianjin Medical University General Hospital, Tianjin 300052, P.R. China

**Keywords:** apoptosis, retinal ischemia/reperfusion, simvastatin, tumor necrosis factor-α, nuclear factor-κB

## Abstract

Simvastatin, which is widely used in the prevention and treatment of hyperlipidemia-associated diseases, has been reported to enhance the survival of retinal ganglion cells (RGCs) in a model of retinal ischemia/reperfusion (IR) injury. However, the underlying mechanism of the anti-apoptotic effects of simvastatin on the retina have yet to be elucidated. In the present study, rats were treated with simvastatin or saline for 7 days prior to IR via ligation of the right cephalic artery. The results showed that simvastatin prevented the apoptosis of RGCs and cells in the inner nuclear layer. Furthermore, simvastatin regulated the expression of apoptosis-associated proteins. The expression levels of the anti-apoptotic protein B-cell lymphoma-2 were upregulated 4 and 24 h after IR in the simvastatin/IR group compared to those in the saline/IR group. Conversely, the levels of pro-apoptotic protein Bax were downregulated in the simvastatin/IR group compared to those in the saline/IR group. Furthermore, the results of the present study showed for the first time, to the best of our knowledge, that simvastatin decreased IR injury-induced tumor necrosis factor-α (TNF-α) and nuclear factor-κB (NF-κB) expression in the retina. These findings strongly suggested that simvastatin inhibits apoptosis following IR-induced retinal injury by inhibition of the TNF-α/NF-κB pathway. The present study also provided a rationale for developing therapeutic methods to treat IR-induced retinal injury in the clinic.

## Introduction

Retinal ischemia/reperfusion (IR), which is a serious and common clinical problem in patients with visual impairment, can occur in diabetic retinopathy, glaucoma and ocular trauma (1–4). Reperfusion of blood following ischemia can aggravate the damage in the retina through mechanisms including oxidative stress, damage of capillaries and inflammatory responses (1). IR is a common pathological process, which leads to the death of retinal ganglion cells (RGCs) and degeneration of the retina, resulting in loss of vision. At the cellular level, IR induces multi-cascade responses, including neuronal depolarization, calcium imbalance, nitric oxide, energy failure and increased glutamatergic stimulation, leading to oxidative stress (5–7).

IR is known to lead to apoptosis of cells in the retina. Apoptosis, which is a programmed form of cell death, can be induced by various death receptor signaling pathways (8). Quantitative analysis of cell apoptosis in a model of retinal IR has revealed increases in apoptotic cells in various retinal layers. Lam *et al* (9) have tested the apoptosis of cells in the retinal ganglion cell layer (GCL) and the inner nuclear layer (INL) of the retina by using a terminal deoxynucleotidyl trans-ferase-mediated dUTP nick end labeling (TUNEL) staining assay. Their results showed that the number of apoptotic cells increased at 2 h following IR as compared to that in the control group and reached a peak at 24 h. Sellés-Navarro *et al* (10) showed that most periods of IR influenced the proportion of RGC death. In addition, Nickells (11) found that IR can induce the expression of proteins which are involved in apoptotic pathways. Ko *et al* (12) found that simvastatin enhanced the survival of RGCs in the IR group compared to that in the control group. Consistent with these results, Krempler *et al* (13) reported that simvastatin improved the survival of RGCs. To study the pathologic mechanisms in the retina and to assess potential clinical therapeutic approaches, it is required to develop novel strategies for protecting the visual function.

Simvastatin, which is an anti-hyperlipidemic drug, is a 3-hydroxy 3-methylglutaryl coenzyme A reductase inhibitor. It is effective in lowering low-density lipoprotein cholesterol and improving clinical outcomes of patients with cardiovascular conditions (14). Furthermore, simvastatin is effective in the treatment of inflammation by directly or indirectly triggering pro-inflammatory signaling pathways (15). Numerous studies have reported that simvastatin prevented fibrosis in certain types of injured tissues, including articular cartilage, lungs, kidneys, heart and blood vessels (16). In addition, certain studies have demonstrated that simvastatin enhanced the survival of RGCs by regulating the anti-apoptotic protein B-cell lymphoma 2 (Bcl-2) in a model of IR (17,18). Apoptosis is a programmed form of cell death and comprises an intrinsic and an extrinsic pathway. Li *et al* (19) reported that autocrine tumor necrosis factor-α (TNF-α) induced nuclear factor-κB (NF-κB) activation in cancer cells. Another study demonstrated that simvastatin inhibited apoptosis by suppressing the expression of TNF-α/NF-κB in a model of burn injury (20). To the best of our knowledge, the ability of simvastatin to suppress the TNF-α/NF-κB pathway in a model of IR has not been explored to date.

In the present study, rats were pre-treated with saline or simvastatin, and IR was performed via ligation of the right cephalic artery. The effect of simvastatin on the apoptosis of cells in each layer of the retina after IR was examined; furthermore, the expression of apoptosis-associated proteins was examined. The hypothesis that simvastatin prevents apoptosis of retinal cells by regulation of TNF-α/NF-κB pathway was also tested.

## Materials and methods

### Chemicals

Simvastatin was purchased from Hangzhou MSD Pharmaceutical Company (Zhejiang, China). Bcl-2 (polyclonal rabbit anti-rat; Cat. no. 2876), Bcl-2-associated X protein (Bax; polyclonal rabbit anti-rat; Cat. no. 2272), NF-κB (monoclonal rabbit anti-rat; Cat. no. 8242) and GAPDH (monoclonal rabbit anti-rat; Cat. no. 2118) antibodies were obtained from Cell Signaling Technology (Danvers, MA, USA). TNF-α (polyclonal rabbit anti-rat; Cat. no. AAR33) antibody was obtained from AbD Serotec (Kidlington, UK). The horseradish peroxidase-conjugated anti-rabbit immunoglobulin G secondary antibody was purchased from Santa Cruz Biotechnology, Inc. (Cat. no. sc-2004; Santa Cruz, CA, USA). A TUNEL staining kit (Cat. no. ZK-8004) was obtained from Beijing Zhongshan Jinqiao Biological Technology (Beijing, China). A RNA extraction kit (RP55000) and RT-PCR kit were obtained from Beijing Baitaike Biological Technology (Cat. no. RP7101; Beijing, China).

### Establishment of a rat model of IR-induced retinal injury

Seventy-two male Sprague-Dawley rats (age, 4 weeks; weight, 240–300 g) were purchased from the Military Medical Academy of China (Beijing, China). The rats were kept in specific pathogen-free conditions throughout the duration of the study with a 12-h light/dark cycle and room temperature (21–23°C). This study was approved by the Ethics Committee of Tianjin Medical University. The rats were fed a standard diet (23% protein) and allowed free access to water (6 rats/cage). Rats were divided into three groups (24 rats/group): Control group, saline/IR group, and simvastatin/IR group. Rats in the control group received an identical treatment to those in the experimental group but without the treatment and the ligation of the right cephalic artery. Rats in the simvastatin- or saline-treated group were treated with simvastatin (20 mg/kg) or saline for 7 days (1 ml/once/day) by intragastric administration before IR injury. Each of the groups was divided into three sub-groups, which were examined at 4 and 24 h after injury, respectively. IR was induced as previously described (23). Briefly, rats were injected with sodium pentobarbital (60 mg/kg body weight i.p.) for anesthesia. The neck skin and subcutaneous muscles were cut, and the right cephalic artery was ligated with a 3-0 suture line. One hour later, the ligation of the right cephalic artery and suture was loosened.

### RNA extraction and reverse transcription quantitative polymerase chain reaction (RT-qPCR)

Total RNA was extracted using the RNA extract kit according to the manufacturer’s instructions (Cat. no. RP55000; Beijing Baitaike Biological Technology). RNA was reverse transcribed into cDNA with MMLV reverse transcriptase (Promega, Madison, MI, USA). The mRNA levels of Bcl-2 (Primer: 5′-ACGAGTGGGATACTGGAGATG-3′ and 5′-TAGCGACGAGAGAAGTCATCC-3′), Bax (Primer: 5′-GCGATGAACTGGACAACAACAT-3′ and 5′-TAGCAAAGTAGAAAAGGGCAACC-3′), TNF-α (Primer: 5′-GTCTACTGAACTTCGGGGTGATCG-3′ and 5′-GAGATAGCAAATCGGCTGACGGTG-3′) and NF-κB (Primer: 5′-CGGCCAAGCTTAAGATCTGCCGAGTAAA-3′ and 5′-GCTGCTCTAGAGAACACAATGGCCACTTGCC-3′) were analyzed by RT-qPCR. The mRNA levels were normalized to β-actin RNA expression (Primer: 5′-CCCATCTATGAGGGTTACGC-3′ and 5′-TTTAATGTCACGCACGATTTC-3′). qPCR was performed on an ABI 7500 sequence detector system (Applied Biosystem, Grand Island, NY, USA) in 50 ml reaction volumes using TransStart Green qPCR SuperMix Kit (Cat. no. AQ101-01; TransGen Biotech, Beijing, China). The 2^−ΔΔCt^ method was used to determine the relative mRNA fold changes. Assays were performed at least 3 times.

### Immunohistochemistry

Formalin-fixed, paraffin-embedded retinal sections were stained with hematoxylineosin (H&E). Immunohistochemistry was performed as previously described (21). Briefly, the staining for Bcl-2 and Bax was performed on rat retinas by using monoclonal antibodies against Bcl-2 and Bax at a dilution of 1:100. The Immunocruz^®^ staining system (Santa Cruz Biotechnology, Inc.) was employed according to the manufacturer’s instructions. For TUNEL staining, retinal sections were stained for apoptotic cells with a TUNEL staining detection kit according to the manufacturer’s instructions. Apoptotic cells were assessed in six fields at a magnification of x400.

### Western blot analysis

Western blot analysis was performed as previously described (22). Briefly, the retina was ground in a homogenizer and proteins were extracted according to the manufacturer’s instructions. Twenty micrograms of protein were electrophoresed by 10% SDS-PAGE (Cat. no. 456-8033; Bio-Rad Laboratories, Inc, Philadelphia, PA, USA) and transferred onto a polyvinylidene difluoride (PVDF) membrane (Cat. no. 8242; EMD Millipore, Bedford, MA, USA). After blocking with 5% bovine serum albumin (Cat. no. BP9703-100; Fisher Scientific, Pittsburgh, PA, USA), the PVDF membrane was immunoblotted with TNF-α antibody (1:1,000), NF-κB antibody (1:1,000), and GAPDH antibody (1:1,000) overnight at 4°C. The membranes were then incubated with horseradish peroxidase-conjugated anti-rabbit immunoglobulin G secondary antibody (1:10,000) for 1 h at room temperature. The membranes were then washed for 30 min by TBST (Cat. no. 7647-14-5; Fisher Scientific). The membranes were exposed to FluorChem (Cat. no. 92-14095-00; ProteinSimple Research, San Jose, CA, USA). All western blot analyses were repeated at least three times.

### Statistical analysis

SPSS 17.0 statistical software (SPSS, Inc., Chicago, IL, USA) was used for all statistical analyses. Values are expressed as the mean ± standard deviation. Differences between groups were analyzed using Student’s paired t-test or one-way analysis of variance, as appropriate. P<0.05 was considered to indicate a statistically significant difference.

## Results

### Simvastatin protects the retina from IR-induced injury

**Figure 1:**
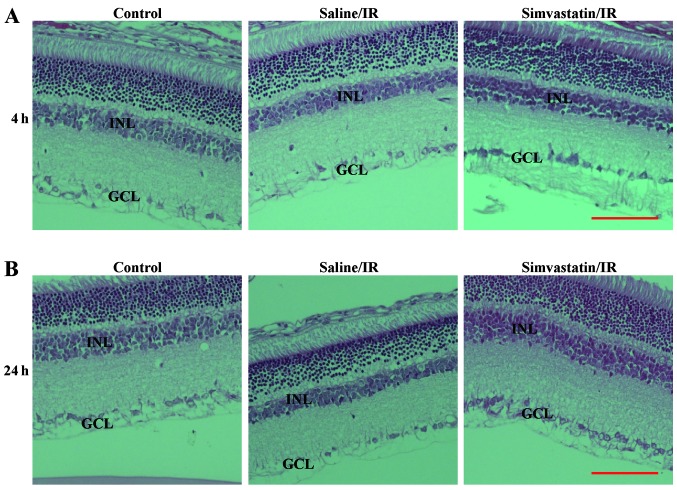
Hematoxylin and eosin staining of the retina in an IR model. Histochemicel images of retina of the control group, saline/IR group and simvastatin/IR group at (A) 4 h and (B) 24 h post-IR (scale bars, 100 µm). IR, ischemia/reperfusion; GCL, ganglion cell layer; INL, inner nuclear layer.

To determine the establishment of the model of IR-induced retinal injury, H&E staining was performed. In the control group, cells in every layer appeared normal and the staining was even at 4 and 24 h post-IR. There was no significant difference in the morphology of the retina in the saline/IR group at 4 h compared with that in the control group (Fig. 1A). A severe effect on retinal morphology was observed at the 24 h time point in the RGCs in the saline/IR group compared with the control group. This included the compression of the INL and a decreased number of cells in RGCs from saline/IR group (Fig. 1B). The number of RGCs was increased in the simvastatin/IR group compared to that in the saline/IR group (Fig. 1B). In addition, the changes in INL thickness in the retina were not obvious in the simvastatin/IR group compared to those in the control group (Fig. 1B). These results suggested that IR injury decreased the number of RGCs in the saline/IR group and the thickness of the INL, while simvastatin protected the retina from IR-induced injury.

### Simvastatin reduces apoptosis of retina cells following IR-induced injury

**Figure 2:**
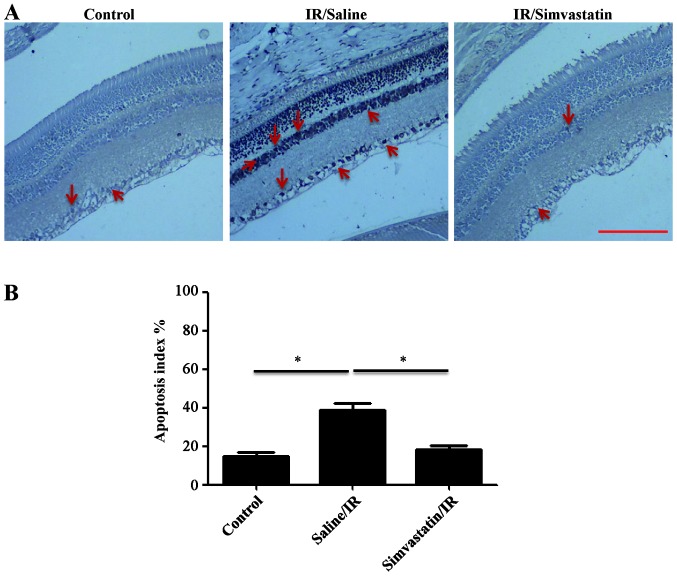
Effects of simvastatin on apoptosis in the retina of an (A) TUNEL staining images of control group, saline/IR group and simvastatin/IR group (scale bars, 100 µm). (B) Bar graph showing the quantified results of the TUNEL staining analysis, expressed as the apoptotic rate. Values are expressed as the mean ± standard deviation (n=6). Red arrows indicate apoptotic cells. ^*^P<0.05. TUNEL, terminal deoxynucleotidyl transferase-mediated dUTP nick end labeling; IR, ischemia/reperfusion.

To further investigate whether simvastatin protects cells of the retina from apoptosis, TUNEL staining was performed. The number of apoptotic cells was slightly increased in the GCL and INL of the retina in the saline/IR group (data not shown) compared to that in the control group at 4 h. Furthermore, TUNEL staining indicated a significant increase in the number of apoptotic cells in the saline/IR group (39%) compared to that in the control group (14.9%) at 24 h (Fig. 2). Simvastatin rescued the number of apoptotic cells in the retina compared to that in the saline/IR group at 24 h (P<0.05) (Fig. 2). This result suggested that IR injury increased the number of apoptotic cells in the GCL and INL, and that simvastatin had anti-apoptotic effects on the cells in the GCL and INL.

### Simvastatin exerts anti-apoptotic effects by regulating the expression of Bcl-2 and Bax

**Figure 3:**
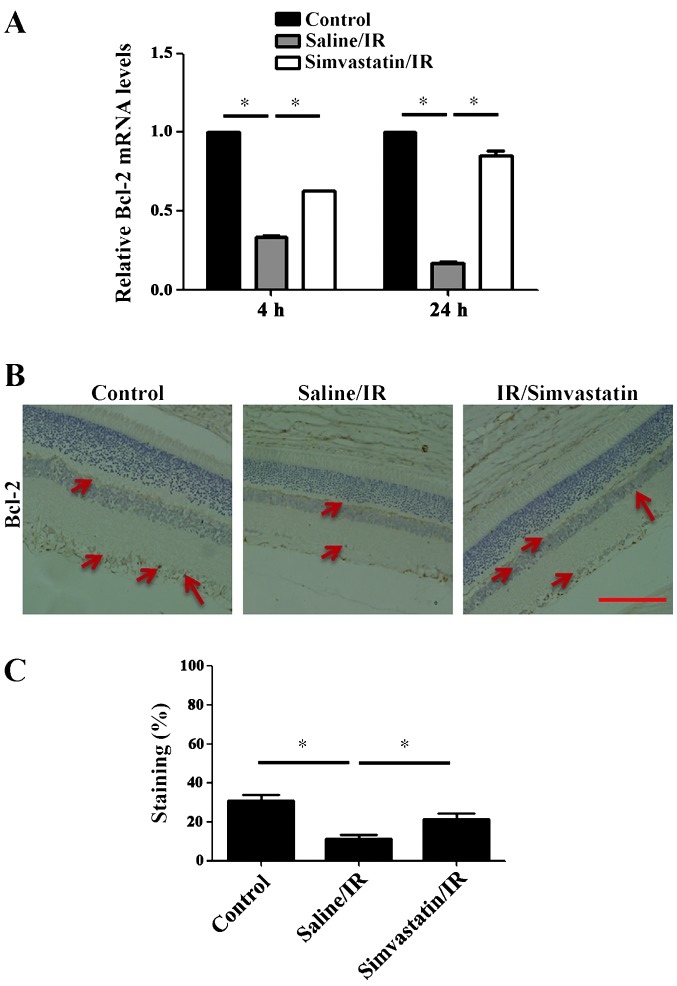
Simvastatin rescues the expression of Bcl-2 in a rat model of IR. (A) Reverse transcription polymerase chain reaction analysis of Bcl-2 mRNA levels in control group, saline/IR group and simvastatin/IR group. (B) Immunohistochemical analysis of Bcl-2 levels in the retina of rats in the control group, saline/IR group and simvastatin/IR group at 24 h (scale bars, 100 µm). (C) Bar graph showing the Bcl-2 levels obtained by quantification of immunohistochemical images. Values are expressed as the mean ± standard deviation (n=6). Red arrows indicate Bcl-2-expressing cells. ^*^P<0.05. IR, ischemia/reperfusion; Bcl-2, B-cell lymphoma 2.

**Figure 4:**
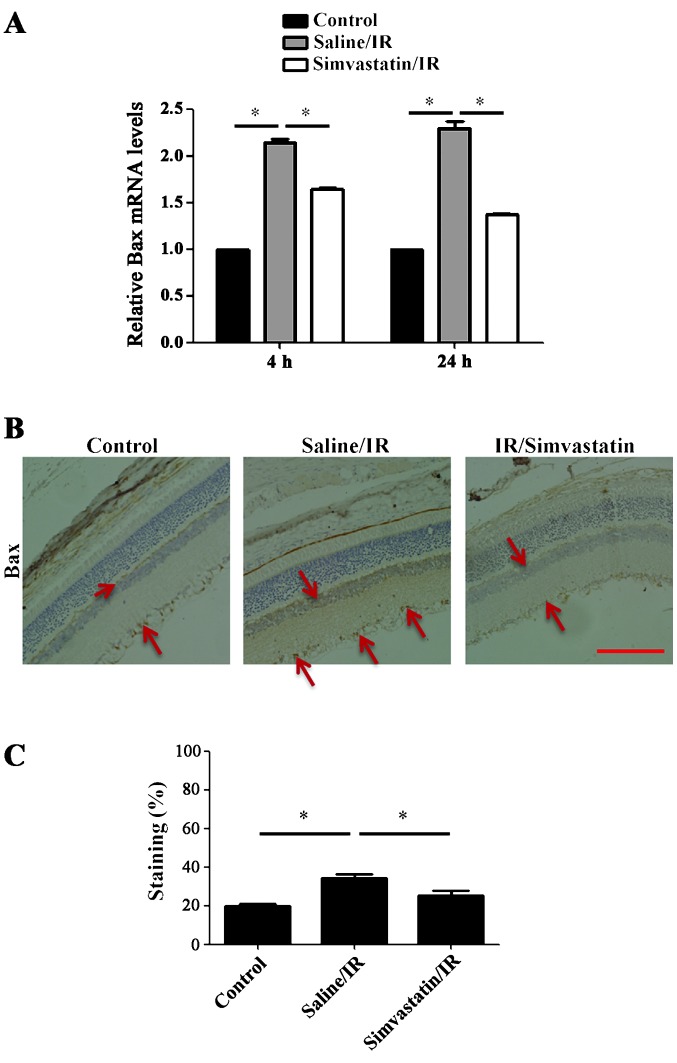
Simvastatin inhibits the induction of Bax in a rat model of IR. (A) Reverse transcription polymerase chain reaction analysis of Bax mRNA levels in control group, saline/IR group and simvastatin/IR group. (B) Immunohistochemical analysis of Bax levels in the retina from rats in the control group, saline/IR group and simvastatin/IR group at 24 h (scale bars, 100 µm). (C) Bar graph showing Bax levels obtained by quantification of immunohistochemical images. Values are expressed as the mean ± standard deviation (n=6). Red arrows indicate Bax-expressing cells. ^*^P<0.05. IR, ischemia/reperfusion; Bax, B-cell lymphoma 2-associated X protein.

Since previous studies suggested that simvastatin upregulated the expression of Bcl-2 protein but not levels of Bax in the retina, these apoptosis-associated proteins were assessed in the present study. RT-qPCR analysis showed that mRNA levels of Bcl-2 were significantly reduced in the retina of the saline/IR group compared to those in the control group at 4 and 24 h (Fig. 3A). Furthermore, levels of Bcl-2 mRNA were increased in the simvastatin-treated group compared to those in the saline/IR group. Consistent with the mRNA levels, immunohistochemical analysis showed that Bcl-2 protein levels in the saline/IR group were slightly reduced at 4 h (data not shown), and were significantly reduced at 24 h, which was significantly attenuated by pre-treatment with simvastatin (P<0.05) (Fig. 3B and C). Conversely, the expression of Bax was significantly increased in the saline/IR group at 4 and 24 h, which was significantly attenuated in by treatment with simvastatin (P<0.05) (Fig. 4). These results demonstrated that simvastatin attenuated the IR-induced increase in the expression of anti-apoptotic protein Bcl-2 and the decrease in the expression of pro-apoptotic protein Bax, therefore inhibiting IR-induced apoptosis in the retina of rats.

### Simvastatin reduces IR-induced increases in TNF-α and NF-κB expression in the retina

**Figure 5:**
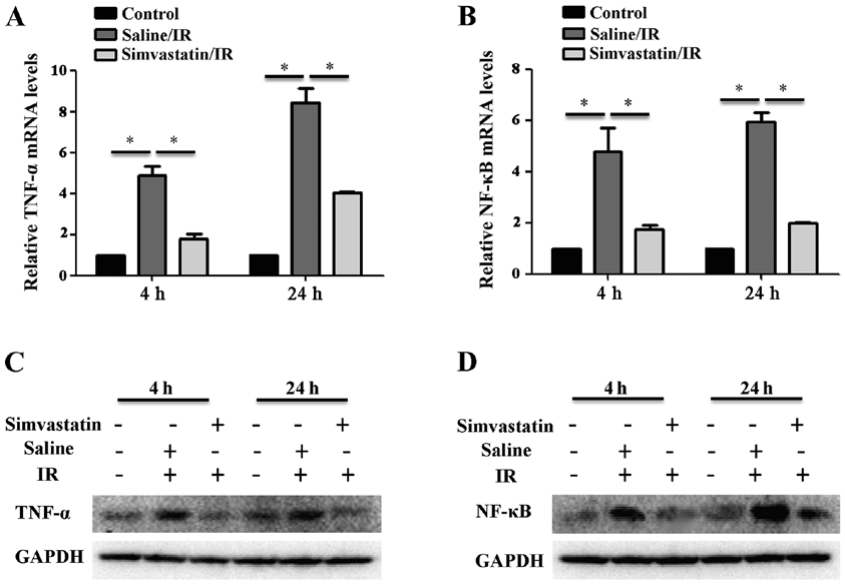
Effects of simvastatin on the levels of TNF-α and NF-κB in the retina of a rat model of IR. Reverse transcription polymerase chain analysis of (A) TNF-α and (B) NF-κB mRNA levels in the retina of rats in the control group, saline/IR group and simvastatin/IR group. Values are expressed as the mean ± standard deviation (n=6). ^*^P<0.05. Western blot analysis of (C) TNF-α and (D) NF-κB protein levels in the retina of rats in the control group, saline/IR group and simvastatin/IR group. IR, ischemia/reperfusion; TNF-α, tumor necrosis factor-α; NF-κB, nuclear factor-κB.

To further examine the mechanism of action of simvastatin on the regulation of apoptosis in the retina, the levels of two important pro-inflammatory cytokines, TNF-α and NF-κB, which are involved in apoptosis signaling, were assessed. Levels of TNF-α and NF-κB mRNA were increased in the saline/IR group compared to those in the control group at the 4- and 24-h time-points (Fig. 5A and B). Simvastatin was able to reduce the levels of TNF-α by ~3-fold compared to those in the saline/IR group (P<0.05) (Fig. 5A). Similarly, >2-fold decreases were observed in the levels of NF-κB in the simvastatin/IR group compared to those in the saline/IR group (P<0.05) (Fig. 5B). To further confirm the results of the RT-qPCR analysis, protein levels were assessed by western blot analysis. The results indicated that the protein levels of TNF-α and NF-κB were upregulated in the saline/IR group compared to those in the control group, while simvastatin obviously decreased the protein levels of TNF-α and NF-κB at 4 and 24 h post-IR (Fig. 5C and D). These findings demonstrated that simvastatin reduced the IR injury-induced increases in TNF-α and NF-κB levels.

## Discussion

The present study investigated the potential effects of simvastatin on IR-induced retinal injury in rats. The results showed that simvastatin prevented the apoptosis of RGCs and cells in the INL after retinal IR injury. In addition, pre-treatment with simvastatin rescued the IR-incuded decreases in the expression of the anti-apoptotic protein Bcl-2 and downregulated the IR-induced increases in the expression of the pro-apoptotic protein Bax. Furthermore, it was shown that the underlying mechanism of the protective effect of simvastatin is mediated, at least in part, via the attenuation of IR-induced increases in the inflammatory cytokines TNF-α and NF-κB.

Retinal ischemia is caused by diabetic retinopathy, retinopathy of prematurity, ischemic optic neuropathy and glaucoma (1,21). IR-induced retinal injury can lead to tissue edema, energy-dependent dysfunction and death of RCGs (13). Death of RCGs occurs via numerous mechanisms, including reduced retinal perfusion, oxidative stress, NO or excitatory amino acids. To reduce the severe effects of retinal ischemia, it is important to discover novel clinical treatment strategies.

The present study found that IR-induced retinal injury resulted in a 2.62-fold increase of apoptotic cells in the GCL and INL at 24 h. Of note, significantly fewer apoptotic RGCs and cells in the INL were present in the simvastatin/IR group compared to those in the saline/IR group. This result demonstrated that simvastatin prevented apoptosis of retinal cells in a rat model of IR-induced retinal injury. A previous studies reported that simvastatin protected retinal neurons from IR-induced injury via the regulation of apoptosis-associated proteins (12). However, to the best of our knowledge, the underlying mechanism of the anti-apoptotic effects of simvastatin has not yet been investigated.

Apoptosis, a programmed cell death, has been identified in retina following ischemia-induced injury. When the apoptosis signaling pathway is activated, Bax, a pro-apoptotic protein, is translocated to the mitochondria. Cytochrome C release leads to the initiation of the apoptotic cascade, which can be prevented by the anti-apoptotic protein Bcl-2 via regulation of Bax. The balance of Bcl-2 and Bax has an important role in the apoptotic process. Ko *et al* (12) reported that simvastatin upregulated Bcl-2 expression in early IR injury. Consistent with these results, the present study showed that pre-treatment with simvastatin significantly rescued Bcl-2 mRNA levels at 4 and 24 h and obviously increased protein levels of Bcl-2 at 24 h following IR injury. Ko *et al* (12) further reported that treatment with simvastatin 24 h after IR did not significantly affect the levels of Bax. By contrast, the results of the present study showed that simvastatin significantly attenuated increases in Bax levels in the retina following IR. This discrepancy in results may be due to differences in the time-point of simvastatin treatment. The findings of the present study demonstrated that simvastatin regulated the expression of apoptosis-associated proteins.

Previous studies have found that simvastatin inhibited the activities of numerous important pro-inflammatory factors, including TNF-α and NF-κB (16,23). TNF-α can activate the NF-κB signaling pathway, which is involved in apoptosis (24). Zhao *et al* (20) found that TNF-α and NF-κB knockout mice showed reduced apoptosis in the spleen in a severe burn injury model. However, to date, it has not been reported whether simvastatin affects the IR injury-induced levels of TNF-α and NF-κB. Therefore, the present study assessed the levels of TNF-α and NF-κB in rats with IR-induced retinal injury, which had been pre-treated with simvastatin. The results showed, for the first time, to the best of our knowledge, that simvastatin attenuated the increases in the expression of TNF-α and NF-κB in the retina of rats following IR model. This indicated that simvastatin inhibited IR-induced apoptosis by suppressing the activity of TNF-α/NF-κB. However, it remains elusive whether simvastatin regulates TNF-α and NF-κB directly or indirectly. In addition, further studies are required to investigate the adequate dose for humans to achieve similar effects to those in rats.

In conclusion, the present study has showed that IR-induced retinal injury induced significant levels of apoptosis via affecting the expression of Bcl-2 and Bax and increasing TNF-α/NF-κB expression. Simvastatin prevented the apoptosis of retinal cells by attenuating the increases in the expression of TNF-α/NF-κB and restoring the balance of the apoptotic signaling protiens Bcl-2 and Bax. The results of the present study strongly suggested that simvastatin is a potential therapeutic for the treatment of IR-induced retinal injury.
